# Internalization of Met Requires the Co-Receptor CD44v6 and Its Link to ERM Proteins

**DOI:** 10.1371/journal.pone.0062357

**Published:** 2013-04-23

**Authors:** Susanne Hasenauer, Dieter Malinger, David Koschut, Giuseppina Pace, Alexandra Matzke, Anja von Au, Véronique Orian-Rousseau

**Affiliations:** Karlsruhe Institute of Toxicology, Institute for Toxicology and Genetics, Karlsruhe, Germany; H.Lee Moffitt Cancer Center & Research Institute, United States of America

## Abstract

Receptor Tyrosine Kinases (RTKs) are involved in many cellular processes and play a major role in the control of cell fate. For these reasons, RTK activation is maintained under tight control. Met is an essential RTK that induces proliferation, differentiation, migration, survival and branching morphogenesis. Deregulation of Met by overexpression, amplification or lack of effective degradation leads to cancer and metastasis. We have shown that Met relies on CD44v6 for its activation and for signaling in several cancer cell lines and also in primary cells. In this paper, we show that internalization of Met is dependent on CD44v6 and the binding of Ezrin to the CD44v6 cytoplasmic domain. Both CD44v6 and Met are co-internalized upon Hepatocyte Growth Factor induction suggesting that Met-induced signaling from the endosomes relies on its collaboration with CD44v6 and the link to the cytoskeleton provided by ERM proteins.

## Introduction

Receptor tyrosine kinases (RTKs) orchestrate several cellular processes such as proliferation, differentiation, migration or survival. As a consequence the activation process must be kept under tight control. In physiological conditions, RTK activation is only transient. Upon ligand stimulation at the cell membrane, RTKs are internalized via endocytosis and are then either recycled to the cell membrane or traffic to different endosomal compartments before degradation. The trafficking route ends in the lysosome, where the degradation process takes place.

It is now well accepted that the internalization process is not simply a mean to remove cell surface receptors from the membrane. RTKs such as Epidermal Growth Factor Receptors (EGF-Rs) accumulate in the endosomes when activated [1]. EGF-R localized at the endosomal membrane can then meet other signaling partners and mediate specific cellular responses. Indeed, Ras can be activated either at the cell surface or at the level of endosomes whereas activation of phospholipase C (PLC-γ) occurs exclusively at the cell surface [2], [3]. Signaling from the endosomes might simply be a mean to either amplify a specific signaling pathway or to diversify the cellular responses. Localized signaling might also help to induce polarized cellular response. Such an example is shown in the case of the Hepatocyte Growth Factor (HGF) activation of Rac via the RTK Met. Activation of Rac by its Guanidine Exchange Factor (GEF), Tiam1 occurs on early endosomes and depends on Rab5, a small GTPase that is essential for endocytosis [4]. Recycling of activated Rac to the membrane is then necessary for actin remodeling.

Met, a major RTK that controls development and tumorigenesis gets transiently activated after HGF induction and like most other RTKs is then internalized via a clathrin-dependent mode [5]. However, the internalization process and subsequent trafficking is not completely unraveled. Upon activation, Met is ubiquitinated through the ubiquitin ligase Cbl, a process that seems not to be required for the internalization step itself as a mutant of Met in the Cbl binding site, namely Y1003, is still internalized and accumulates in endosomal membranes where it promotes sustained Mitogen-Activated Protein Kinase (MAPK) activation [6]. In the case of other RTKs, such as the Fibroblast Growth Factor Receptor (FGF-R) [7] and EGF-R [8], mutations in the major ubiquitination sites also do not affect internalization (for review, see [9]).

More and more evidence demonstrate that RTKs are not only activated through ligand binding and that the activation process is much more complex [10]. One way to increase the panel of cellular responses consists in collaborating with several partners. The association of FGF-R with N-cadherin or with E-cadherin is such an example. In the presence of N-cadherin, FGF-R internalization is reduced and the accumulation of activated FGF-R at the cell membrane leads to transformation (reviewed in [11]). In contrast, both FGF-R and E-cadherin are co-internalized and transported to the nucleus where cell-cycle progression is induced.

It is very likely that the various partners recruited by RTKs also influence the internalization process thereby controlling the cellular outcome. A prime example of co-receptor control over RTK trafficking, is given by Vascular Endothelial Growth Factor-2 (VEGFR-2), the most prominent receptor in angiogenesis. Association of VEGFR-2 with its co-receptor neuropilin-1 (reviewed in [12]) promotes recycling through Rab11 vesicles consequently allowing p38 MAPKinase activation, an essential pathway for sprouting angiogenesis [13].

Met has been shown to collaborate with several partners such as integrin β4 [14] or plexins [15]. The best-characterized Met partners are members of the CD44 family of transmembrane glycoproteins containing the exon v6 (abbreviated as CD44v6) (reviewed in [16]). CD44v6 plays a dual role in the Met activation process. The ectodomain of CD44v6 is required for the activation step itself, most probably through binding of HGF and presentation to the receptor. In addition, the cytoplasmic domain of CD44v6 connected to ERM proteins (Ezrin, Radixin, Moesin) and to the cytoskeleton via the ERM actin-binding domain, is essential for activation of Ras via its GEF SOS [17], [18].

This molecular interaction between CD44v6 and Met is relevant for several biological processes. Indeed, the entry of the bacteria *Listeria monocytogenes* into HeLa cells relies on the interplay between Met, CD44v6, Ezrin and the cytoskeleton [19]. In vivo, the angiogenic process [20] and the metastatic propensity of pancreatic carcinoma cells (Matzke et al., unpublished) also rely on the association of the Met-CD44v6 signalosome to the cytoskeleton.

In this paper, using immunofluorescence and biochemical methods, we show that Met and CD44v6 are co-internalized and that the cytoplasmic domain of CD44v6 is required for internalization in addition to the binding of ERM proteins to the cytoskeleton. Indeed, there is a lack of internalization of Met in the presence of a cytoplasmic deletion mutant of CD44v6 or an Ezrin mutant deleted in the actin-binding domain. Blocking of CD44v6 by means of peptides abrogate the internalization process of Met completely. These data indicate that CD44v6 does not only exert its function as a co-receptor for Met at the membrane but is also associated to Met on the trafficking routes where Met is able to induce specific signaling pathways.

## Results and Discussion

### 1) HGF Leads to Co-internalization of CD44v6 and Met

**Figure 1:**
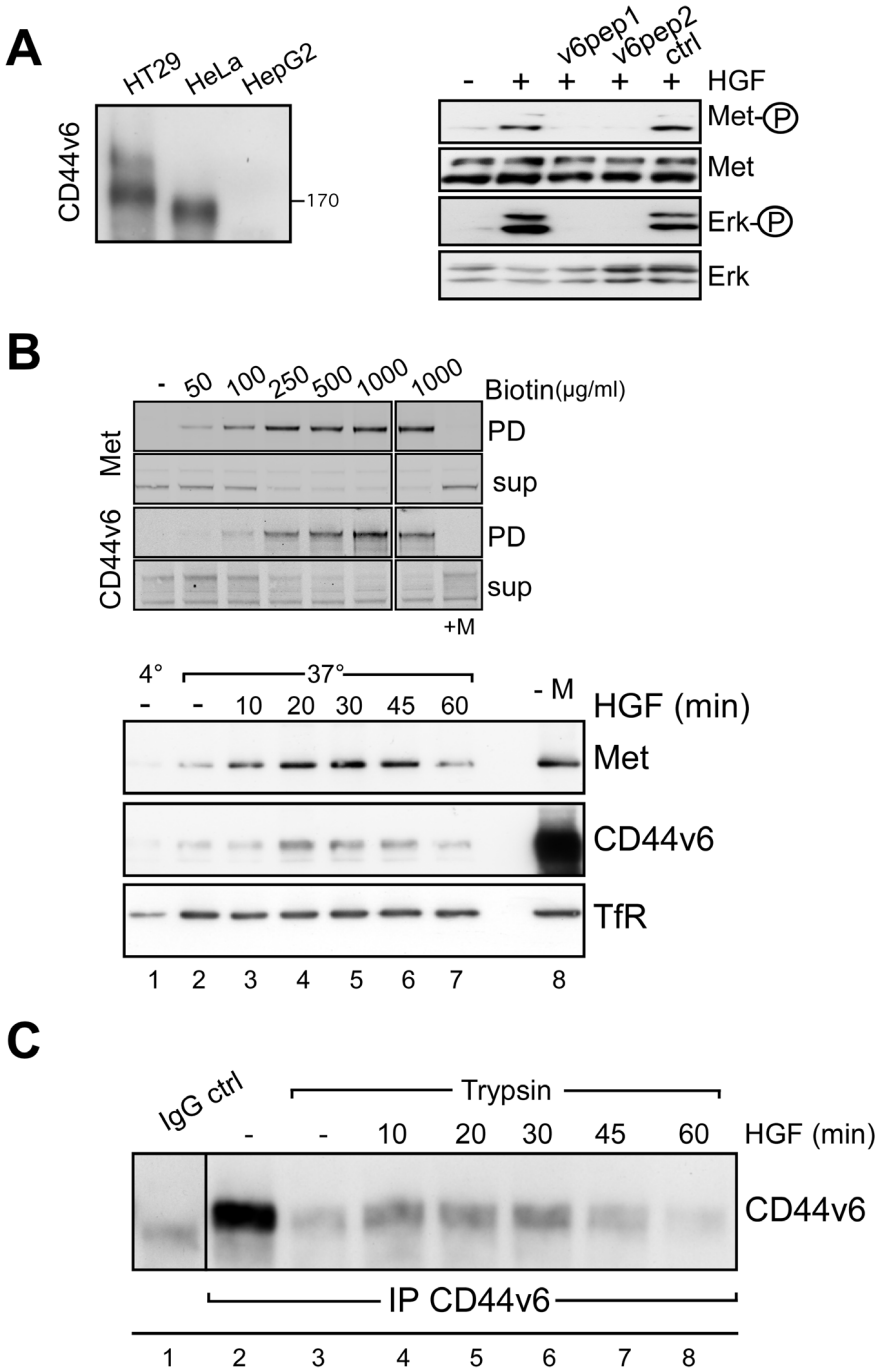
CD44v6 is internalized upon HGF induction. (**A**) Left side: Lysates of HT29, HeLa and HepG2 cells were immunoprecipitated with the CD44v6 antibody (VFF18) and subjected to SDS-PAGE. The blots were probed with the same antibody. Apparent MW is indicated. Right side: starved HeLa cells were induced for 5 min with HGF (25 ng/ml) and Met and Erk phosphorylation was determined as described in Material and Methods. Where indicated cells were pre-treated with CD44v6 peptides (v6pep1: 5mer, v6pep2: 14mer) or a control peptide (see Material and Methods). Met and Erk hybridization was used as loading controls. The experiment was repeated at least three times. (**B**) Top: The optimal biotin concentration for efficient biotinylation was determined (50–1000 µg/ml). Serum starved cells were biotinylated for 15 minutes on ice and biotinylated proteins were pulled down using a NeutrAvidin resin. Both fractions, the pull down (PD) and the supernatant (sup) were subjected to Western Blot analysis using Met (25H2) and CD44v6 antibodies as indicated. The MESNA treatment (last lane) was tested with the highest concentration of biotin (1 mg/ml). Below: Serum starved cells were biotinylated (0,5 mg/ml), induced with HGF (50 ng/ml) and treated with MESNA (Material and Methods). After lysis, internalized (still biotinylated) proteins were pulled down with a NeutrAvidin resin. The fractions were subjected to Western Blot analysis and Met, CD44v6 and the Transferrin receptor (TfR) were detected with the respective antibodies (Material and Methods). The first sample (lane 1) was kept at 4°C. –M: the cells were not treated with MESNA. (**C**) HGF-induced internalization of surface proteins detected by the trypsin assay. Serum starved cells were induced by HGF (25 ng/ml) for the indicated times and then cooled down on ice followed by a treatment with trypsin for 30 minutes (Materials and Methods). FCS was added to stop the trypsin treatment and the cells were then lysed. CD44v6 was immunoprecipitated and detected by Western Blot analysis. First lane: immunoprecipitation with IgG.

In order to study the possible role of CD44v6 in Met internalization we chose HeLa cells in which the internalization process of Met has extensively been studied [21], [22]. In these cells a high molecular weight band at about 170 kD can be detected by means of a CD44v6 specific antibody (Figure 1A left). HT29 cells in which the co-receptor function of CD44v6 for Met was intensively examined were used as a positive control [17]. The HepG2 cells that do not express any CD44 isoform [23] were used as a negative control. The dependency of Met activation towards CD44v6 has been established in several primary and cancer cells [17]. Similarly to HT29 cells [24] both the phosphorylation of Met and Erk could be blocked by means of CD44v6 peptides in HeLa cells (Figure 1A right and in [19]). Indeed, pre-incubation of HeLa cells with a CD44v6 5mer (v6pep1) or 14mer (v6pep2) peptide drastically reduced the phosphorylation of Met and its downstream target Erk.

To measure Met internalization we used a modified internalization assay [25]. This assay consists in the labeling of surface proteins using a biotin harboring a disulfide bond that can be cleaved off by the reducing agent sodium 2-mercaptoethanesulfonic acid (MESNA). HeLa cells were labeled with biotin at 4°C. At this temperature, the RTKs bind their ligand but do not internalize and accumulate in clathrin-coated pits [26], [27], [28]. The optimal amount of biotin was determined by a titration experiment (Figure 1B). Cells were lysed and the amount of labeled proteins was determined by precipitation with a NeutrAvidin-coupled resin followed by an SDS-PAGE. 500 µg/ml of biotin was enough to label both Met and CD44v6 proteins to saturation under our experimental conditions (Figure 1B upper part). Upon incubation with MESNA (last lane) no signal could be detected in the precipitate indicating that the MESNA treatment removed biotin with 100% efficiency. In Figure 1B lower part, the cells were labeled with biotin as described above and then induced with HGF for the indicated time points (lanes 3–7) at 37°C. The cells were then treated with MESNA three times for 10 minutes at 4°C. Receptors that were internalized were protected from the MESNA treatment (the cells are not permeable to MESNA) and could be pulled down from the lysates by means of a NeutrAvidin-coupled resin. Lane 1 represents a control lane where the cells were incubated at 4°C in the absence of HGF. The total amount of biotinylated surface proteins pulled down by NeutrAvidin in the lysate in the absence of HGF and of MESNA is shown in lane 8. At 37°C, in the absence of HGF, a basal level of Met internalization could be observed (lane 2). The internalization of Met was induced upon incubation with HGF for 10 minutes and reached a maximum at 30 minutes. After 45 minutes of incubation with HGF the signal started to decrease and was strongly reduced at 60 minutes most probably due to Met degradation. Upon induction with HGF, CD44v6 was also internalized. The time-course of CD44v6 internalization was similar to the one described for Met. Indeed, in the case of CD44v6 as well, the maximum of internalization was observed around 20 to 30 minutes and a decrease could be seen thereafter. At 60 minutes, degradation of CD44v6 was also observed as in the case of Met. Unlike CD44v6 and Met, the trafficking of the constitutively recycling transferrin receptor (TfR) [29] was not affected by incubation with HGF as shown in Figure 1B lower part. The TfR blot also indicates that the amount of proteins in each sample was very similar and serves as an internal control.

In order to confirm that CD44v6 is indeed internalized upon HGF induction, we used another biochemical method that like the MESNA experiment also enables to distinguish between internalized versus cell surface proteins (Figure 1C). This technique makes use of trypsin, an enzyme that can digest proteins such as CD44v6. If a trypsin digest follows the induction by HGF all cell surface proteins are cleaved while the internalized proteins are protected from the extracellular trypsin and remain intact. The cell lysis was followed by an immunoprecipitation with a CD44v6 antibody. A basal level of internalization was detected in the absence of HGF (lane 3). After 10 to 30 minutes (lanes 4–6) of induction with HGF the internalization of CD44v6 seemed to be maximal similarly to the MESNA experiment. From 45 minutes on (lanes 7,8), the signal decreased indicating degradation. Lane 2 shows an immunoprecipitate of CD44v6 as compared to the unspecific IgG control immunoprecipitation (lane 1).

**Figure 2:**
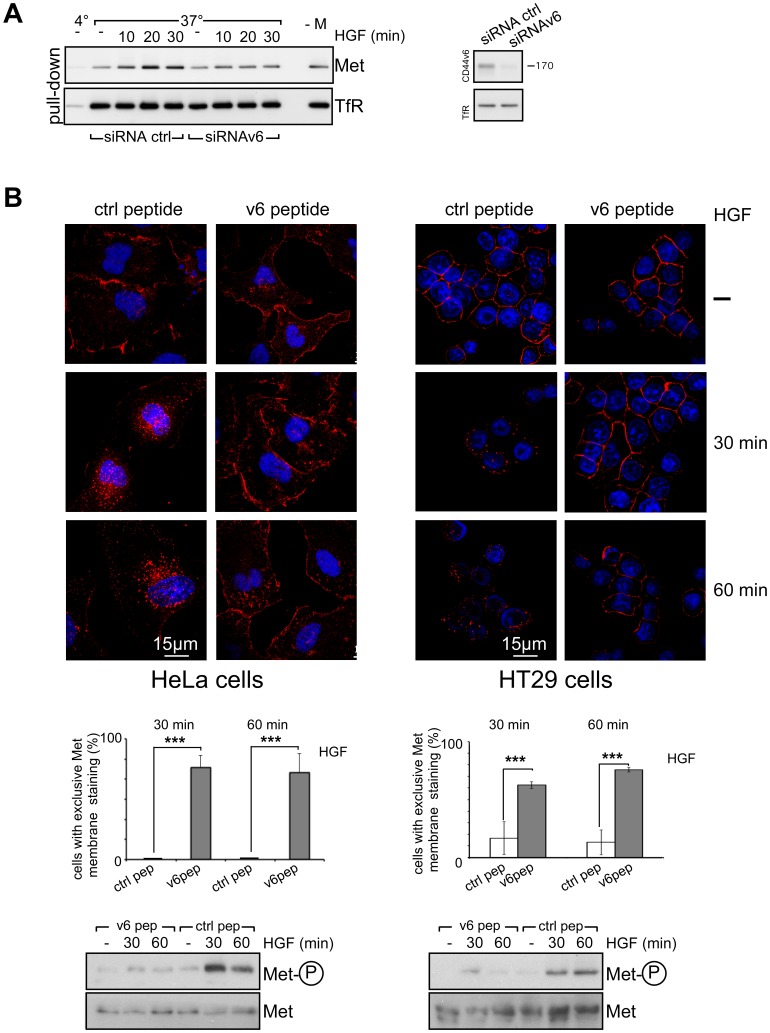
CD44v6 controls Met internalization. (**A**) Left side: HeLa cells were transfected either with a pool of control siRNAs or a mixture of two different CD44v6 specific siRNAs and starved for 24 hours (Material and Methods). The cells were biotinylated (0,5 mg/ml), induced with HGF (50 ng/ml) and treated with MESNA. After cell lysis, proteins were pulled down with a NeutrAvidin resin and subjected to Western Blot analysis with Met or the Transferrin receptor (TfR) antibodies. For the first sample the cells were kept at 4°C. –M: the cells were not treated with MESNA. Right side: Western Blot analysis of cell lysates of ctrl siRNAs and CD44v6 siRNAs transfected HeLa cells using the CD44v6 and the TfR antibodies. (**B**) Starved HeLa cells respectively HT29 cells were incubated with the v6 peptide or a control peptide for 10 minutes at 37°C and then induced with 25 ng/ml of HGF for the indicated time points. Cells were then either lysed and the lysates were subjected to Western Blot analysis for phospho-Met and Met (below) or cells were fixed, permeabilized and stained for Met with specific antibodies (red) (above). Nuclei were stained with Dapi and images were taken with a confocal microscope (Leica SPE) using a 63× objective. The quantification of three independent experiments (n = 40) is shown. The percentage of cells with Met exclusively located at the plasma membrane was calculated for each time point. Student´s t test: ***p<0,001.

Since CD44v6 is required for Met activation, repression of CD44v6 expression should also have an effect on Met internalization. To test this possibility we performed a MESNA experiment in HeLa cells transfected with a pool of control siRNA or a mixture of two siRNAs against CD44v6 (Figure 2A). Upon downregulation of CD44v6 expression (Figure 2A, right panel), the internalization of Met was reduced to background level (Figure 2A, left panel). The internalization of the Transferrin Receptor (TfR) was not at all affected by inhibition of CD44v6 expression by siRNA. Thus the internalization of Met appears strictly dependent on CD44v6 most likely due to its role in the activation of the Met receptor.

Met-induced angiogenesis [20] and Met-induced metastasis of pancreatic carcinoma cell (Matzke et al, unpublished data) can be inhibited by a CD44v6 specific peptide that interferes with the CD44v6 co-receptor function. To show that this co-receptor function of CD44v6 for Met regulates the internalization of Met, we pre-incubated HeLa cells with a v6 peptide or a control peptide prior to induction with HGF and followed the internalization process of Met using confocal microscopy (Figure 2B left). In both cases (control peptide and v6 peptide treated cells), Met was located at the membrane in the absence of HGF. In the control peptide treated cells, Met internalization followed the kinetic shown in Figure 1B (Figure 2B left). Indeed, strong dotted staining suggesting endosomal localization could be observed at 30 and 60 minutes of HGF induction. The quantification indicates that most cells have lost the membrane staining. In stark contrast, Met stayed at the membrane and almost no internalization was detected upon pre-incubation of HeLa cells with the CD44v6 peptide (Figure 2B left) that was also shown to abrogate Met activation in Figures 1A (right side) and Figure 2B below. A similar experiment was performed with the HT29 cells (Figure 2B right). In the control peptide treated cells, Met was internalized after HGF induction. Thus the control peptide has no inhibitory effect. In contrast, the CD44v6 peptide drastically decreased Met internalization and Met was detected at the membrane at all time points. The quantification (Figure 2B below) clearly indicates that most cells treated with the CD44v6 peptide retained Met at the membrane. A Western Blot analysis shows the inhibition of Met activation by the CD44v6 peptides (Figure 2B below).

**Figure 3:**
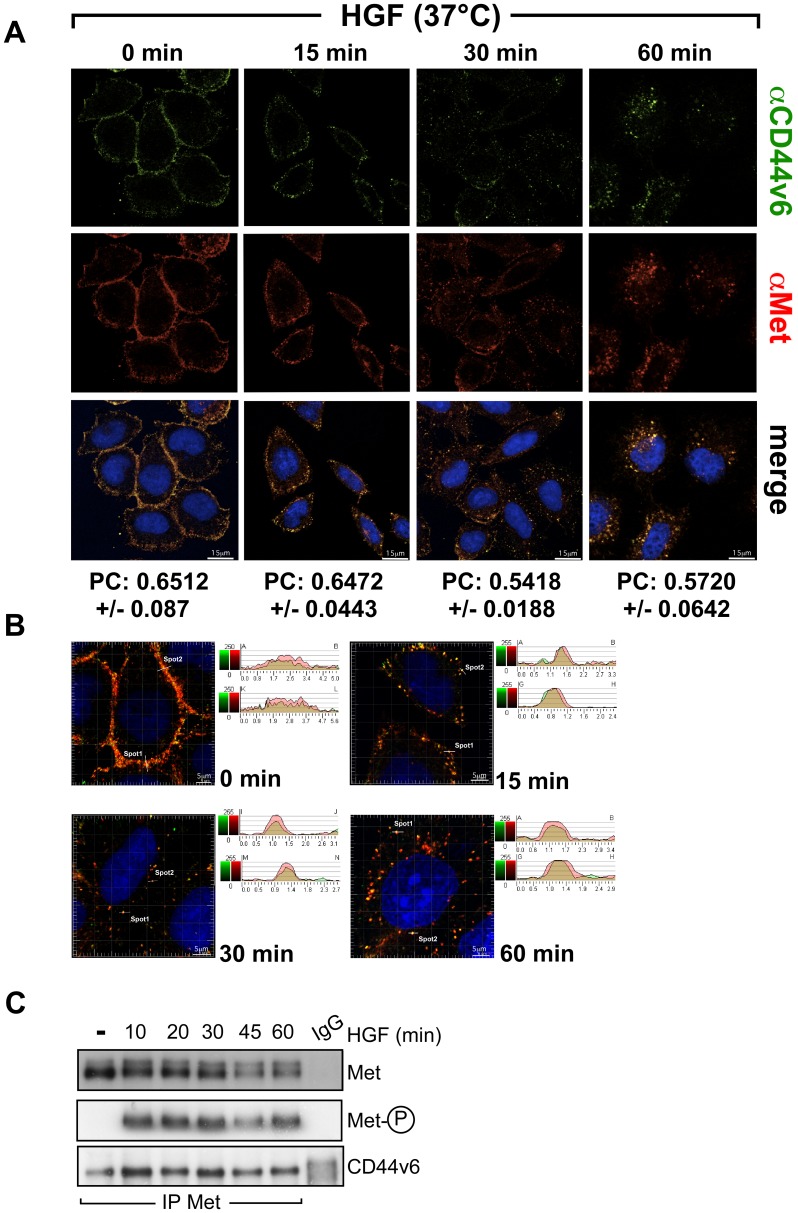
Co-localization of Met and CD44v6 in internal compartments after HGF stimulation. (**A**) Confocal images of endogenous Met and CD44v6 at different time points after HGF-induction. HeLa cells were serum starved, treated for 2 hours with 50 µM cycloheximide, incubated at 4°C for 1 hour with HGF (25 ng/ml) (cold start) and then shifted to 37°C for the indicated times. After fixation cells were stained for Met (red, AF276) and CD44v6 (green, VFF18) and nuclei (blue, Draq5) and imaged with the confocal microscope (LSM510) with a 63× objective (Material and Methods). For each time point, the mean Pearson Coefficient of 4–6 images was calculated using the Imaris software. Scale bar = 15 µm (**B**) Intensity profiles of the emission wavelengths of the Met and CD44v6 signals along lines drawn across the plasma membrane or vesicles containing both Met and CD44v6. The intensity of the respective wavelengths is plotted against the distance in µm. (**C**) Co-immunoprecipitation of CD44v6 and Met upon HGF induction. Serum-starved HeLa cells were induced with HGF (50 ng/ml) for the indicated time periods and Met was immunoprecipitated. The precipitate was subjected to Western Blot analysis and the membrane was blotted for Met, phospho-Met and CD44v6. The experiment was repeated at least three times.

In order to study whether the cellular distribution pattern of CD44v6 overlaps with the one observed for Met, we used confocal microscopy (Figure 3A). HeLa cells were treated with cycloheximide in order to block de novo synthesis of proteins prior to the induction with HGF. The cells were treated with HGF for one hour at 4°C to allow binding but not internalization. This procedure is referred to as a cold start. Receptors were then allowed to internalize upon temperature increase to 37°C. Directly after the cold start both CD44v6 and Met were exclusively located at the plasma membrane. 15 minutes after the 37°C switch, the membrane staining for both CD44v6 and Met became less prominent and spots could be detected close to the membrane. 30 minutes post temperature switch, accumulation of Met and CD44v6 was detected in internal compartments. At 60 minutes, both proteins could be detected close to the nucleus.

Co-localization was quantified by measuring the Pearson Coefficient (PC) [30] using the Imaris Software (see Figure 3A). The mean values of the PC for 6 images are indicated. In all cases the merged picture and the PC indicate that Met and its co-receptor CD44v6 are co-localized and traffic together upon HGF induction.

The intensity profiles were also measured for all time points and showed a perfect match for the red and the green channels (Figure. 3B). The width of the peaks at 15, 30 and 60 minutes corresponds to the size of an endosome (approximately 0.5–1 µm).

To further confirm the possible association between CD44v6 and Met after induction with HGF, endogenous Met was immunoprecipitated from HeLa cells and immunoblotted with a CD44v6 antibody at different time points (Figure 3C). The association between Met and CD44v6 was inducible by HGF as already described [17] and was maximal after 10 minutes of induction with HGF at 37°C. Complexes between CD44v6 and Met could even be detected at 60 minutes. Even after that time the Met receptor remained activated.

**Figure 4:**
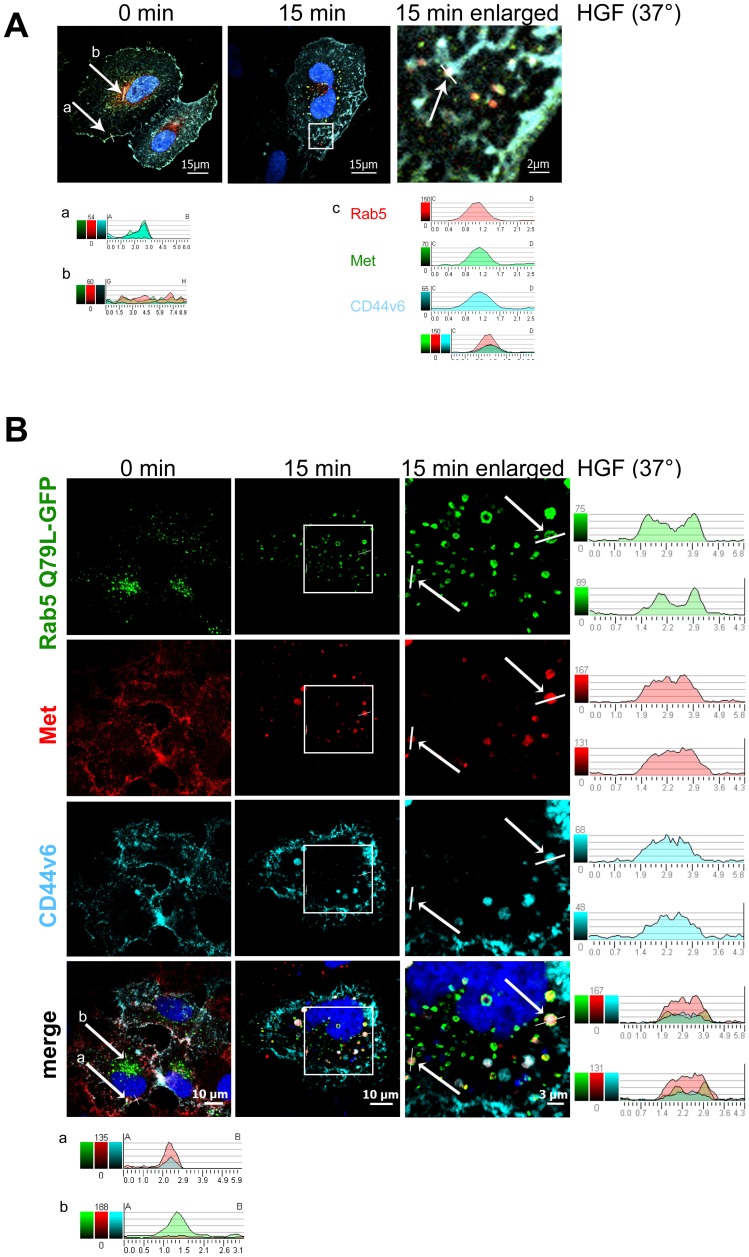
Colocalization of Met and CD44v6 in Rab5 positive endosomes upon HGF stimulation. (**A**) HeLa cells were transfected with mRFP-Rab5 wild type and human CD44v6. After 24 hours the cells were serum starved and treated with 50 µM cycloheximide for 2 hours. Cells were stimulated with 25 ng/ml HGF for 1hour on ice (cold start) and subsequently shifted to 37°C for the indicated time. Cells were fixed, permeabilized, and stained for Met (green) and CD44v6 (cyan) with specific antibodies (Materials and Methods). Nuclei were stained with Dapi (blue) and images were taken with a confocal microscope (Leica SPE) using a 63× objective. The last image is an enlargement of the square indicated in the middle image. Intensity profiles of the respective emission wavelengths of the mRFP-Rab5, Met, and CD44v6 signals were measured along lines drawn across the plasma membrane (a) or across vesicles (b,c). (**B**) HeLa cells were transfected with a constitutive active Rab5-GFP construct (Rab5 Q79L-GFP) and a human CD44v6 construct (see Materials and Methods) and treated as in A. Rab5 Q79L-GFP (green), Met (red), CD44v6 (cyan), Dapi (blue). Intensity profiles of the respective emission wavelengths of Rab5 Q79L-GFP, Met and CD44v6 are shown on the right side for the indicated endosomes at 15 minutes. Intensity profiles corresponding to the plasma membrane (a) and to an endosome (b) are included for the 0 minute time point (below).

The first intracellular location of RTKs upon uptake is the early endosome (reviewed in [31]). Rab5 controls the internalization steps from the membrane to early endosomes and can be used as a marker of early endosomes [32]. To visualize the trafficking of CD44v6 and Met to early endosomes we transfected a tagged wild-type Rab5 construct (mRFP Rab5, red) and a human CD44v6 construct in HeLa cells (Figure 4A). In this experiment, cells were submitted to a cold start and fixed immediately (0 min) or shifted to 37°C for 15 minutes. Cells were stained for CD44v6 (cyan) and for endogenous Met (green). Confocal microscopy pictures revealed that the three molecules were not co-localized directly after the cold start (Figure 4A, a and b). However, co-localization was visible 15 minutes post temperature switch as indicated by the intensity profiles (Figure 4A,c).

In order to enhance the detection of Rab5 positive endosomes, we made use of a Rab5 constitutively active GFP construct (Rab5-Q79L-GFP) that has been used in other studies and behaves similarly to wild-type Rab5 [13]. Co-transfection of HeLa cells with this activated Rab5-GFP version and a human CD44v6 construct was performed (Figure. 4B). Met (red) and CD44v6 (cyan) were detected with the respective specific antibodies and Alexa Fluor-labeled secondary antibodies. The merged picture showing the distribution of these three proteins and the intensity profiles revealed that both Met and CD44v6 are present in Rab5 positive endosomes 15 minutes post temperature switch. Directly after the cold start (0 min), no co-localization was observed and both Met and CD44v6 were located at the membrane whereas Rab5 was located on endosomes (Figure 4B, a,b). Colocalization of Met and endogenous Rab5 (Figure S1) indicate that the wild-type Rab5 and the constitutively active Rab5 constructs behave like endogenous Rab5.

Taken together, our data suggest that CD44v6 is recruited to Met upon HGF induction and traffics together with Met to internal compartments where at least part of the Met receptor remains activated. Furthermore, CD44v6 even controls the internalization process of Met itself.

It was previously shown that Met signaling also emanates from the endosomal compartment and not exclusively from the plasma membrane [25], [33], [34], [35]. Indeed, trafficking to endosomes but not to perinuclear areas seems to be essential for HGF-induced Erk phosphorylation and is controlled by PKC [35]. In contrast, the Met-activated STAT3 signaling depends on the perinuclear localization of Met through a microtubules and PKCα-dependent process [34]. CD44v6 might stay associated to Met in endosomal compartments in order to promote signaling from these internal locations.

### 2) The CD44v6 Cytoplasmic Domain and Ezrin are Required for Met Internalization

**Figure 5:**
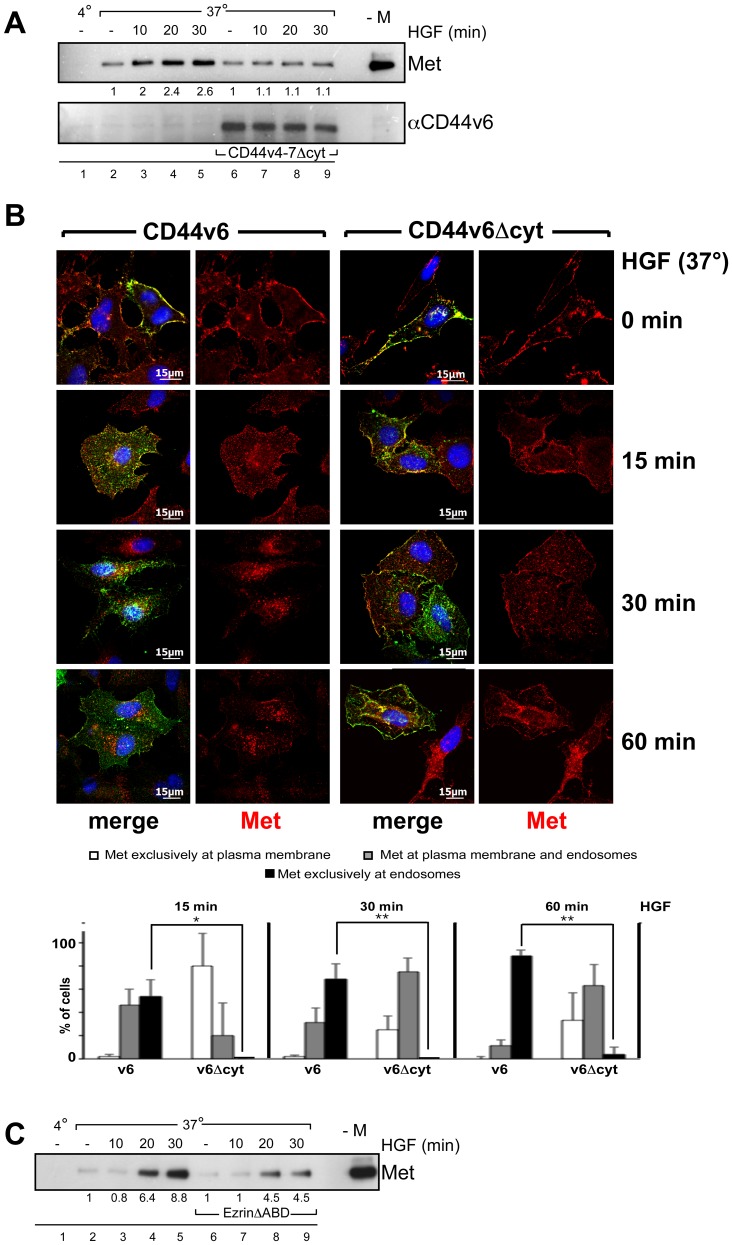
The link between CD44v6 and the cytoskeleton through Ezrin is required for Met internalization. (**A**) HeLa cells were transfected with a rat CD44v4-7 construct deleted from the cytoplasmic domain (CD44v4-7Δcyt) or a control vector. The kinetic of Met internalization upon HGF induction was measured in a MESNA experiment (Material and Methods). In the first sample cells were kept at 4°C. –M refers to a sample obtained from cells that were not treated with MESNA. The Western Blot analysis was performed with a Met specific antibody and the rat specific CD44v6 antibody 1.1ASML (Material and Methods). (**B**) HeLa cells were transfected either with human CD44v6 wild type or with human CD44v6 tailless constructs as indicated. After 24 hours cells were serum starved, treated with 50 µM cycloheximide for 2 hours prior to HGF-induction, stimulated with 25 ng/ml HGF for 1 hour on ice (cold start) and incubated at 37°C for the indicated time periods. Cells were then fixed, stained, and imaged using a confocal microscope (Leica SPE) with a 63× objective. Met (red), CD44v6 wild type (green), CD44v6 tailless (green), Dapi (blue). Scale bar = 15 µm. The quantification of three independent experiments (n = 20) is shown. The percentage of transfected cells with Met exclusively located on endosomes or exclusively at the plasma membrane or on both was calculated for each time point. Values are the means of three independent experiments. Student´s t test: *p<0,05 and **p<0,01. (**C**) HeLa cells were transfected with an Ezrin construct deleted from the actin-binding domain (EzrinΔABD) or a control vector. The cells were serum starved for 24 hours followed by biotinylation (0,5 mg/ml), induced with HGF (50 ng/ml) and treated with MESNA. Upon cell lysis proteins were pulled down by NeutrAvidin beads. The precipitates were subjected to Western Blot analysis for Met and CD44v6. For the first sample the cell were kept at 4°C. –M: cells were not treated with MESNA. The experiment was repeated at least three times.

The cytoplasmic domain of CD44v6 is involved in Met signaling through its binding to ERM proteins that in turn bind to the cytoskeleton. This link between the CD44v6 cytoplasmic domain, ERMs and the cytoskeleton is however not required for the activation of Met per se. In order to examine the contribution of the CD44v6 cytoplasmic domain in internalization, we transfected HeLa cells with a rat CD44 construct containing exon v6 and deleted from the cytoplasmic domain (CD44v4-7Δcyt) and performed a MESNA internalization assay (Figure 5A). This truncated rat protein has been shown to allow Met activation but not signal transduction to Erk in human cells [18]. Moreover it allows discriminating between endogenous and transfected CD44 proteins and identifying transfected cells by rat-specific CD44v6 antibodies. In the case of the control vector (lanes 2–5), the pattern of internalization of Met followed the one that was already shown in Figure 1B. Indeed, from 10 to 30 minutes of HGF induction, the amount of Met that was internalized increased drastically as compared to the sample that was left untreated. In contrast, in the presence of a CD44v4-7Δcyt protein (lanes 6–9), the internalization of Met was reduced to background level.

We then used confocal microscopy to follow Met distribution in HeLa cells transfected either with a human CD44v6 full-length construct or a human CD44v6Δcyt construct (Figure 5B). In this case, the experimental procedure included a cold start. After one hour of incubation with HGF at 4°C, Met could only be detected at the membrane in both the CD44v6 full-length and the CD44v6 mutant transfected cells. 15, 30, and 60 minutes post temperature switch at 37°C, the progressive internalization of Met was observed in the CD44v6 full-length transfected cells. Dotted structures were detected close to the membrane at 15 minutes post temperature switch at 37°C. At 30 and 60 minutes post temperature switch at 37°C, the punctate structures were closer to the nucleus. In total contrast, in the mutant CD44v6Δcyt transfected cells, part of Met seemed to be blocked at the membrane at all time points and remained co-localized with the truncated CD44v6 protein. To quantify the effect of the tailless CD44v6 mutant we counted cells that either had Met exclusively located at the plasma membrane or where Met was found on the membrane as well as on endosomes or where Met was exclusively on endosomes (graph in Figure 5B). The progressive internalization in cells transfected with the CD44v6 full-length construct could be best seen when looking at the percentage of cells with exclusive endosomal Met. The lack of internalization in cells transfected with the mutant CD44v6Δcyt is also seen in the graph. Indeed, the percentage of cells showing an exclusive endosomal staining is extremely low whereas cells with exclusive Met staining at the plasma membrane are even found after 60 min.

As we expected from previous experiments regarding the co-receptor function of CD44 isoforms for Met [17], the experiments in Figure 5A and B demonstrate that the rat CD44v4-7Δcyt and the human CD44v6Δcyt both inhibit Met internalization in HeLa cells. To further confirm this point the experiment of Figure 5B was repeated using the rat CD44v4-7Δcyt construct instead of the human CD44v6Δcyt (see Figure S2). In that case as well similar results were obtained confirming that these two constructs behave similarly.

The CD44v6 cytoplasmic domain is able to bind to ERM proteins through a basic stretch of amino acids (for review see [36]). ERM proteins in turn are able to bind the cytoskeleton through a C-terminal actin-binding domain. Removal of 29 amino acids in the C-terminal domain inhibits binding of ERM proteins to the actin cytoskeleton [37]. To test whether such a network including the CD44v6 cytoplasmic domain, Ezrin and the cytoskeleton is as well involved in the internalization process of Met we analyzed whether a mutation in the actin-binding domain acts in a dominant negative fashion. In the presence of an Ezrin protein deleted from its actin-binding domain (EzrinΔABD), the internalization process of Met was reduced in comparison to cells transfected with the control vector (Figure 5C). After 20 minutes of HGF induction, the internalization of Met was reduced to 30%. After 30 minutes, around 50% less internalization took place.

These results suggest that Ezrin through binding to the cytoskeleton is a major player in the CD44v6 control over Met internalization. A role of Ezrin and CD44v6 in internalization might be linked to the movement of Clathrin Coated Vesicles (CCVs) from the membrane. Actin assembly via activation of N-WASP or Arp2/3 has been shown to play a role in propulsion of endocytic vesicles [38]. Several evidences suggest that Ezrin can indirectly promote de novo actin polymerization in addition to its F-actin anchoring function. On purified phagosomes Ezrin induces F-actin assembly by recruiting the N-WASP-Arp2/3 machinery [39]. In the membrane of these phagosomes partners of Ezrin like the CD44 proteins are also present. It is therefore possible that CD44 and Ezrin play an important role in intracellular trafficking at early stages by directly influencing the CCVs movement. Moreover, Ezrin has already been detected on endosomes [40].

Another possible role of ERM proteins such as Ezrin in endocytosis might be the regulation of PIP2 concentrations. The enrichment and turn over of PIP2 are closely linked to the assembly and dis-assembly of endocytic vesicles. There is direct evidence that Ezrin binds PIP2 through its N-terminal domain [41] and the Ezrin-FERM domain binding to PIP2 might influence the concentration of PIP2.

Besides the influence of Ezrin on the endocytic machinery, it might also directly control Met turn over. Indeed, it was recently reported that Ezrin upon ubiquitination by the ubiquitin ligase WWP1 is able to control Met stability [42]. The ubiquitination of Ezrin does not however target it for degradation.

**Figure 6:**
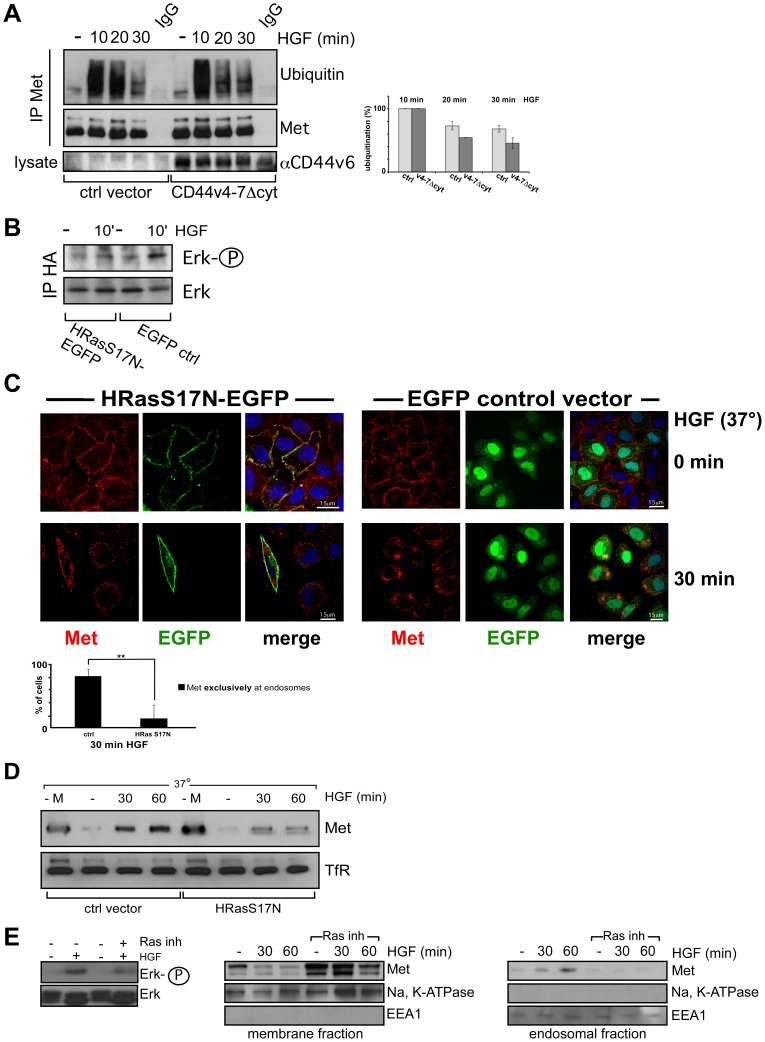
HRas signaling is required for HGF-induced Met internalization. (**A**) HGF-dependent ubiquitination of Met in HeLa cells transfected with a tailless mutant of CD44. HeLa cells were transfected as indicated with either the rat CD44v4-7Δcyt construct or a control vector as described in Material and Methods. 24 hours after transfection cells were starved and incubated with HGF (25 ng/ml) at 37°C for the indicated time periods. The Met immunoprecipitates were loaded for SDS-PAGE and subjected to Western Blot analysis using antibodies against ubiquitin and Met (Materials and Methods). For quantification, the intensities of the bands corresponding to three independent experiments were used. (**B**) Constitutively inactive Ras (HRasS17N) blocks HGF-induced Erk-activation. HeLa cells were co-transfected with HA-Erk and either the constitutively inactive Ras (HRasS17N-EGFP) or the control vector. After 24 hours cells were serum starved and induced with HGF (25 ng/ml) for 10 minutes or left untreated. HA-Erk was immunoprecipitated with a HA-antibody and blotted for phospho-Erk and Erk. (**C**) Confocal images of endogenous Met after HGF-induction in HeLa cells transfected with a Ras dominant negative mutant (HRasS17N) or a control vector. HeLa cells were transfected as indicated either with the HRasS17N-EGFP construct or with a control vector (Material and Methods). After 24 hours cells were serum starved, treated with 50 µM cycloheximide for 2 hours, then incubated with HGF for 1 hour on ice (cold start) and subsequently shifted to 37°C for the indicated time period. Cells were then fixed, stained, and imaged using a confocal microscope (Leica SPE) with a 63× objective. Met (red), HRasS17N-EGFP (green), EGFP (green), Dapi (blue). Scale bar = 15 µm. The quantification of three independent experiments (n = 20) is shown. For quantification the percentage of transfected cells with Met exclusively located on endosomes was calculated. Student´s t test: **p<0,01. (**D**) HeLa cells were transfected with the HRasS17N construct or a control vector. The kinetic of Met internalization upon HGF induction was measured in a MESNA internalization assay. - M refers to a sample obtained from cells that were not treated with MESNA. The Western Blot analysis was performed with a Met specific antibody and the TfR antibody. (**E**) Membrane and endosomal fractions were blotted against Met. Na+/K+ ATPase is used as a control for the membrane fraction (middle). EEA1 is used as a control for the endosomal fraction (right). Left: Phosphorylation of Erk is blocked in the presence of the Ras inhibitor (farnesyl thiosalicylic acid).

Met itself is also ubiquitinated upon HGF induction. The ubiquitination of Met seems however not to be involved in internalization per se but rather in the targeting to the lysosomal sorting machinery [6]. Indeed, a mutant form of Met that cannot bind to Cbl and therefore cannot be ubiquitinated is still internalized but is not able to reach the lysosomes. In agreement with this observation Met is still ubiquitinated in the absence of CD44v6 cytoplasmic domain (Figure 6A). In HeLa cells transfected with the CD44v4-7Δcyt mutant, Met was ubiquitinated after 10, 20 and 30 minutes of induction with HGF similarly to what happened in the cells transfected with the control vector (Figure 6A).

However, ubiquitination of proteins required for the first steps of internalization corresponding to the assembly of the clathrin-coated pits (CCPs) might be important. Such proteins are AP2, epsin and Eps15. The latter has been shown to be phosphorylated and ubiquitinated in response to HGF [43].

### 3) Signaling Controls Internalization

Since the CD44v6-Met-Ezrin-cytoskeleton complex has already been shown to be necessary for signaling and more precisely for the activation of Ras via its GEF SOS [18], the involvement of the same players in internalization suggests two possibilities: either signaling is required for internalization or internalization is an independent event. It has previously been reported that signaling regulates endocytosis. Indeed, formation of CCPs and CCVs seems to rely on activation of RTKs signaling at the plasma membrane (for review, see [44]). Furthermore, the activation of Rab5, an early mediator of endocytic trafficking [45], [46] by the GEF RIN1 is directly mediated by Ras [47]. Conversely, EGF-dependent Erk activation was inhibited in HeLa cells transfected with a K44A dynamin mutant [48] suggesting that internalization can also control signaling. However, it seems that the activation of Ras itself might not depend on internalization whereas the step between MEK and Erk does rely on this process (see review see [44]). Another example indicating that internalization influences signaling was recently published in [49]. The Golgi-localized gamma ear-containing Arf-binding protein (CGA3) was shown to control Met recycling and thereby sustained Erk activation and cell migration.

To assess the influence of signaling on the internalization of Met, we inhibited Ras activation by means of a dominant negative Ras mutant, HRasN17GFP [50] (Figure 6). In order to prove that the mutant Ras protein that we used was indeed dominant negative, we transfected this construct in HeLa cells and measured Erk phosphorylation (Figure 6B). As expected, the phosphorylation of Erk was completely blocked confirming that this HRasN17GFP mutant is indeed dominant negative. In HeLa cells this dominant negative Ras mutant interfered with Met internalization (Figure 6C). The quantification (graph in Figure 6C) gives the percentage of cells where Met was exclusively located on endosomes. In the Ras mutant transfected cells the percentage of cells showing an exclusive endosomal staining was marginal at 30 minutes whereas the control vector transfected cells mainly showed an exclusive endosomal staining. The influence of Ras on Met internalization was also measured by means of a MESNA internalization assay (Figure 6D). In the control vector transfected HeLa cells, increasing amounts of internalized Met could be detected after 30 and 60 minutes of HGF induction. In contrast, Met internalization was drastically reduced in cells transfected with the Ras dominant negative construct. The TfR recycling which is not affected by the inhibition of Ras signaling, serves here as a loading control. To further confirm these results, a subcellular fractionation was performed on HeLa cells treated with farnesyl thiosalicylic acid that induce the dislodgement of Ras from the cell membrane rendering it susceptible to degradation [51]. Pre-incubation of cells with this Ras inhibitor blocked Erk phosphorylation as shown in Figure 6E left side. In the control cells, the disappearance of the Met band from the plasma membrane fraction at 30 and 60 min indicated that internalization took place. In contrast, the Met signal could be detected at both time points in the plasma membrane of cells treated with the Ras inhibitor. The Na^+^/K^+^ ATPase is used as a marker for the membrane fraction [52]. In the endosomal fraction, Met accumulated with time in the control cells. No Met could be detected in the endosomal fraction in cells treated with the Ras inhibitor. EEA1 is used as a marker for endosomes [53].

Taken together, these results suggest that the signaling from the Met-CD44v6-Ezrin complex influences the internalization of the Met receptor.

### Conclusions

Genetic evidence reveal an interdependence of CD44 and Met *in vivo* as mice that are *Cd44−/−, Met +/−* die at birth and show a defect in the pre-Bötzinger complex and in the phrenic nerve [54]. The study of the molecular mechanism of action of CD44v6 for Met demonstrates that CD44v6 participates in Met activation and signaling (reviewed in [16]). In this paper, an additional function of CD44v6 for Met in the internalization process strengthens the link between these two molecules.

## Materials and Methods

### Antibodies and Other Reagents

The human CD44 exon v6-specific antibody VFF18 was a gift of Bender (Vienna, Austria). The rat exon v6-specific antibody 1.1ASML has been described [55]. The phospho-Erk (Thr202/Thr204), the phospho-Met (D26) the Met (25H2) and the ubiquitin (P4D1) antibodies were purchased from Cell Signaling Technology (Beverly, UK). The Erk antibody (K-23) was obtained from Santa Cruz (Heidelberg, Germany), the Transferrin Receptor antibody (ab37632) from Abcam (Cambridge, UK) and the human Met antibody (AF276) from R&D Systems (Wiesbaden, Germany). The rabbit IgG and mouse IgG secondary antibodies were from DAKO (Hamburg, Germany). The EEA1 antibody was a kind gift from Michael Clague (Liverpool, UK). The antibody against the Na^+^/K^+^ ATPase was purchased from Abcam (Cambridge, UK). The Ras inhibitor (farnesyl thiosalicylic acid) was purchased from Biomol (Hamburg, Germany). The Rab5 antibody (SPC-168) was bought from StressMarcq. The CD44v6 specific peptides (human 5-mer, human 14-mer) and the control peptides are described [24]. Lyophilized peptides were dissolved in PBS 1% BSA to a stock concentration of 1 µg/µl. Recombinant human HGF (R&D Systems, Wiesbaden, Germany) was activated with 5% fetal calf serum (FCS; PAA Cölbe, Germany) overnight.

### Cell Lines, Cell Culture and Plasmids

The human cervix carcinoma cell line HeLa (American tissue culture collection, ATCC; Wesel, Germany. Accession No: CCL-2), the human colon adenocarcinoma cell line HT29, a gift of A. Zweibaum (Institut National de la Sante et de la Recherche Medicale, France) and the human hepatoma cells HepG2 (ATCC Accession No: HB-8065) were grown in Dulbecco’s modified Eagle’s medium (DMEM; Invitrogen, Karlsruhe, Germany) supplemented with 10% FCS.

The construct expressing an Ezrin mutant deleted in the actin-binding domain (EzrinΔABD) was a gift from M. Arpin (Institute Pasteur, Paris, France). The CD44v4-7Δcyt construct has been described [17]. The HRasS17N-EGFP vector was a gift of Karel Svoboda (Addgene plasmid #18665) and the EGFP vector was from Clontech (Takara Bio Europe/Clontech, St.-Germain-en-Laye, France). The HA-Erk1 construct was a gift from A. Ulrich (Martinsried, Germany). The constitutively active Rab5-GFP construct (Rab5a Q79L GFP) has been described [13] and was kindly provided by Kurt Ballmer-Hofer (PSI, Villingen, Switzerland). The human CD44v6 construct corresponds to the CD44 sequence including only the exon v6 in the extracellular domain. This sequence was cloned into the pEGFP-N2 fusion vector (Clontech). Since the EGFP tag disturbed the function of the protein, the expression of EGFP was repressed upon insertion of a stop-codon behind the CD44v6 sequence. The cloning strategy can be obtained upon request.

### siRNA Inhibition

Cells were transfected with a mixture of two CD44v6-specific siRNAs: v6-1: 5′-AGU AGU ACA ACG GAA GAA ATT-3′; v6-2: 5′-GGA UAU CGC CAAACA CCC ATT-3′ or a pool of non-specific control siRNA. The control siRNAs had a 31%, respectively 47% or 68% GC content: 5′-UAA UGU AUU GGA ACG CAU AUU-3′; 5′-AGG UAG UGU AAU CGC CUU GUU-3′ and 5′-UGC GCU AGG CCU CGG UUG CUU-3′. All siRNAs were obtained from Eurofins MWG GmbH, Ebersberg, Germany. The transfection was performed using Lipofectamine 2000 (Invitrogen, Karlsruhe, Germany). Two rounds of transfection with an interval of 24 h between were performed. 48 hours after the first transfection the cells were serum-starved for 24 hours and then subjected to further treatment.

### Activation of RTKs and Erk

Serum-starved cells were induced with the growth factor HGF (25–50 ng/ml equals 0,3–0,6 nM) at 37°C for the corresponding time periods. Where indicated, cells were treated with a CD44v6 specific peptide or a control peptide (100 ng/ml) for 10 minutes at 37°C prior to the ligand induction. Cells were washed with ice-cold phosphate-buffered saline (PBS). To detect activated Erk and activated Met, cells were lysed in boiling SDS-sample buffer (125 mM TrisHCl pH 6.8; 4% SDS; 20% Glycerol; 0.01% bromophenol blue) containing 100 mM dithiothreitol (DTT) and subjected to Western Blot analysis using antibodies against phosphorylated Erk or phosphorylated Met. After stripping (62.5 mM Tris, pH 6.8, 2% SDS, 0.8% DTT; 1 h; 55°C), the blot was probed with Erk or Met (25H2) antibodies for loading control. Blots were stained using the enhanced chemiluminescence system (ECL, Thermo Fisher Scientific, Schwerte, Germany). Bands in Western Blot analyses were quantified with the program ImageJ (National Institutes of Health).

### Immunoprecipitation

For immunoprecipitation HeLa cells were induced with HGF (50 ng/ml) for the indicated time periods followed by incubation in lysis buffer A (60 mM *n*-octyl -D-glucopyranoside (Sigma-Aldrich, München, Germany), 0.1% SDS (Sigma-Aldrich, München, Germany), 10 mM NaF, 1 mM phenylmethylsulfonyl fluoride, 1 mM Na-orthovanadate, 1 mM aprotinin and 1 mM leupeptin in HBS) for 30 minutes on ice. Upon centrifugation (12 000 g, 20 min) the cleared lysates were incubated with the indicated antibodies at 4°C o/n followed by precipitation with protein A/G agarose beads (Merck, Darmstadt, Germany). The precipitates were washed three times with lysis buffer A and subjected to Western Blot analysis.

### MESNA Internalization Assay

The experiment was performed as described [25]. HeLa cells were grown to 70% confluency and serum starved for 24 hours. Cell surface proteins were biotinylated on ice as described previously [56]. In brief, cells were washed twice with ice-cold PBS and subsequently incubated with 0,4 mg/ml sulfosuccinimidyl-2-(biotinamido) ethyl-1,3-dithiopropionate (EZ-LinkSulfo-NHS-SS-Biotin; Thermo Fisher Scientific, Schwerte, Germany) in 150 mM Na_2_B_4_O_7_ pH 8 for 15 min on ice followed by three washing steps with ice-cold serum-free HBS containing 0.1% BSA and one times HBS containing 0.7 mM CaCl_2_ and 0.5 mM MgCl_2_ (HBS^++^) to quench the reaction. Receptor activation and internalization was induced by shifting the cells to 37°C and adding pre-warmed serum-free medium containing HGF (50 ng/ml). The reaction was stopped at 4°C. After one rinse with HBS^++^ the cells were incubated three times for 10 minutes with ice-cold 100 mM sodium 2-mercaptoethanesulfonic acid (MESNA) in 50 mM Tris-HCl pH 8.6, 100 mM NaCl, 1 mM EDTA, and 0.2% BSA. Then the cells were washed twice with HBS^++^, and residual MESNA was quenched by incubation with ice-cold 120 mM iodoacetamide in HBS^++^ (10 min). The cells were washed two times with ice-cold HBS++ and then incubated with lysis buffer A (see Immunprecipitation) for 10 minutes followed by centrifugation (12 000 g, 7 min). The amount of protein in the supernatant was determined with the BCA kit (Thermo Fisher Scientific, Schwerte, Germany) and equal amounts were incubated overnight at 4°C with 40 µl of NeutrAvidin beads (Thermo Fisher Scientific, Schwerte, Germany). The beads were washed three times with 1% Triton X-100 (Merck, Darmstadt, Germany) in HBS and one time with HBS and prepared for SDS-PAGE.

When necessary the cells were transfected with Lipofectamin (Invitrogen, Karlsruhe, Germany) according to the manufacturer’s protocol (transfection efficiency around 60%). Cells were then incubated with medium for 8 h and serum-starved for 24 h before further processing.

### Trypsin Assay

The experiment is a modified version of the one described in [57]. HeLa cells were grown to 70% confluency and stimulated with HGF for various time periods. Subsequently, cells were shifted to 4°, washed twice with PBS and for 7 minutes in ice-cold 20 mM sodium acetate (pH 3,7). After two washing steps with ice-cold PBS the cells were treated with 0,1% Trypsin (Thermo Fisher Scientific, Schwerte, Germany) in PBS on ice for 30 minutes followed by the addition of FCS. The cells were lysed and handled as described in immunoprecipitation (see above).

### Confocal Microscopy

HeLa cells were seeded on 20 mm glass cover slips (Menzel, Braunschweig, Germany) and serum starved o/n. 2 hours prior to HGF-induction, 50 µM cycloheximide (Sigma-Aldrich, Taufkirchen) was added. Then the cells were incubated in HGF-containing (25 ng/ml) serum-free medium for 1 hour on ice to allow maximal receptor binding (cold start). Subsequently, the cells were shifted to 37°C for the indicated time periods, chilled on ice, washed with ice cold PBS, fixed and permeabilized immediately with methanol (−20°C, 10 min) or fixed with 4% paraformaldehyde for 15 minutes at RT and permeabilized with 0.3% TritonX-100 for 3 minutes at RT. The cells were then incubated for 1 hour in blocking buffer (5% bovine serum albumin, 0.2% Triton X-100, 0.05% Tween 20, PBS) at RT followed by incubation with the respective primary antibodies in blocking buffer (Met (AF276): 10 µg/ml; VFF18: 10 µg/ml; 1.1ASML: 1 µg/ml) o/n at 4°C. Cells were then washed three times for 10 minutes with PBS and incubated with secondary antibodies (for Met: donkey anti goat; for CD44v6: donkey anti mouse) coupled to the Alexa Fluor 488 (green), 546 (red) or 633 (cyan) label (all from Invitrogen, Karlsruhe, Germany), Draq5 (Biostatus, Leicestershire, UK, dilution 1∶1 000) or Dapi (dilution 1∶10 000) (Life Technologies, Darmstadt) in blocking buffer for 1 hour at RT. Samples were mounted with Fluorescence Mounting Medium (Dako, Glostrup, Denmark) and analyzed with a Zeiss LSM510 or Leica SPE laser scanning confocal microscope with a 63× objective. Where indicated cells were transfected with PromoFectin (PromoCell, Heidelberg, Germany) according to the manufacturer’s protocol (transfection efficiency around 60%). 24 hours after transfection, cells were serum starved for 15–18 hours, treated as described above and analyzed by confocal microscopy.

In the case of blocking experiments, cells were pretreated with 100 ng of CD44v6 peptide (14mer) for 10 minutes at 37°C prior to induction with HGF (25 ng/ml) in serum-free medium for the indicated time points. Cells were then washed with ice-cold PBS and handled as described above.

### Ubiquitination Assay

HeLa cells were seeded on 10 cm culture dishes and transfected with the control vector or CD44v4-7Δcyt constructs with PromoFectin according to the manufactureŕs protocol. 24 hours after transfection, the cells were serum starved o/n and induced with 25 ng/ml HGF at 37°C for the indicated time points. Then cells were washed with ice cold PBS and incubated in lysis buffer B (see Met Degradation) containing 2.5 mg/ml N-ethylmaleimide (Sigma-Aldrich, Taufkirchen). Cell lysates were cleared by centrifugation (12 000 g, 10 min) and an aliquot was taken to determine the transfection efficiency. Met was immunoprecipitated by incubation with Met antibodies (25H2) o/n at 4°C and then with Protein A/G agarose beads for 3 hours at 4°C. Beads were washed four times with lysis buffer B and bound proteins were separated by SDS-PAGE and subjected to Western Blot analysis. The Western Blot membrane was then denaturated with 6 M Guanidin-HCl, 20 mM Tris pH 7,4, 1 mM PMSF and 5 mM 2-Mercaptoethanol for 30 minutes at 4°C, washed four times with TBS-T and blocked o/n at 4°C with 5% BSA in TBS-T. The membrane was incubated with the ubiquitin antibody (1∶1 000 dilution) for 1 hour at RT, washed three times for 10 minutes with TBS-T, incubated with HRP-coupled secondary antibody and developed with ECL.

### Subcellular Fractionation

The membrane/endosome fractionation was described earlier [58]. Briefly cells were washed once with ice-cold PBS, scraped and pelleted at 1000 rpm for 5 min. The cell pellet was resuspended in a homogenization buffer (HB) consisting of 250 mM sucrose in 3 mM imidazole pH 7.4 and protease inhibitors (10 mg/ml aprotinin, 1 mg/ml pepstatin, 10 mg/ml leupeptin and 1 mm PMSF) and pelleted again at 2500 rpm for 10 min. The cell pellet was then homogenized in HB supplemented with 1 mM EDTA by seven passages through a 24-gauge needle. Postnuclear supernatant was obtained by centrifugation at 3000 rpm for 10 min. The crude endosomal fraction was then isolated by adjusting the sucrose concentration of the PNS to 40.6% by adding 62% sucrose (1∶1.2, v/v). This fraction was placed in an SW41 ultracentrifuge tube (Beckman), overlaid with 35% sucrose in HB (4,5 ml) and the tube was filled up with HB. The samples were centrifuged at 35000 rpm for 1.5 h at 4°C. Crude endosomal fractions were collected from the interphase betweeen 35% sucrose and HB and the membrane fraction between 40.6% and 35% sucrose. Prior to fractionation, the cells were treated either with a Ras inhibitor (farnesyl thiosalicylic acid) (5 µM) or DMSO O/N and induced with HGF for 30 and 60 minutes.

## Supporting Information

Figure S1 **Colocalization of Met (Red) with endogenous Rab5 (green).**
(TIF)

Figure S2 **The experiment was performed as described in** **Figure 5B** **.** In this case, the cells were transfected with a rat CD44v4-7Δcyt instead of human CD44v6Δcyt.
(TIF)
