# Correction: Internalization of Met Requires the Co-Receptor CD44v6 and Its Link to ERM Proteins

**DOI:** 10.1371/annotation/6e6576eb-77b0-4892-90d4-ab298c00a216

**Published:** 2013-09-17

**Authors:** Susanne Hasenauer, Dieter Malinger, David Koschut, Giuseppina Pace, Alexandra Matzke, Anja von Au, Véronique Orian-Rousseau

The affiliation for all authors was incorrectly given. The correct affiliation is: Karlsruhe Institute of Technology, Institute of Toxicology and Genetics, Karlsruhe, Germany. 

