# Supplementary figures and images for: Internalization of Met Requires the Co-Receptor CD44v6 and Its Link to ERM Proteins
Source: PLoS One. 2013 Apr 23;8(4):e62357. doi: 10.1371/journal.pone.0062357 (PMC3633891; doi:10.1371/journal.pone.0062357)

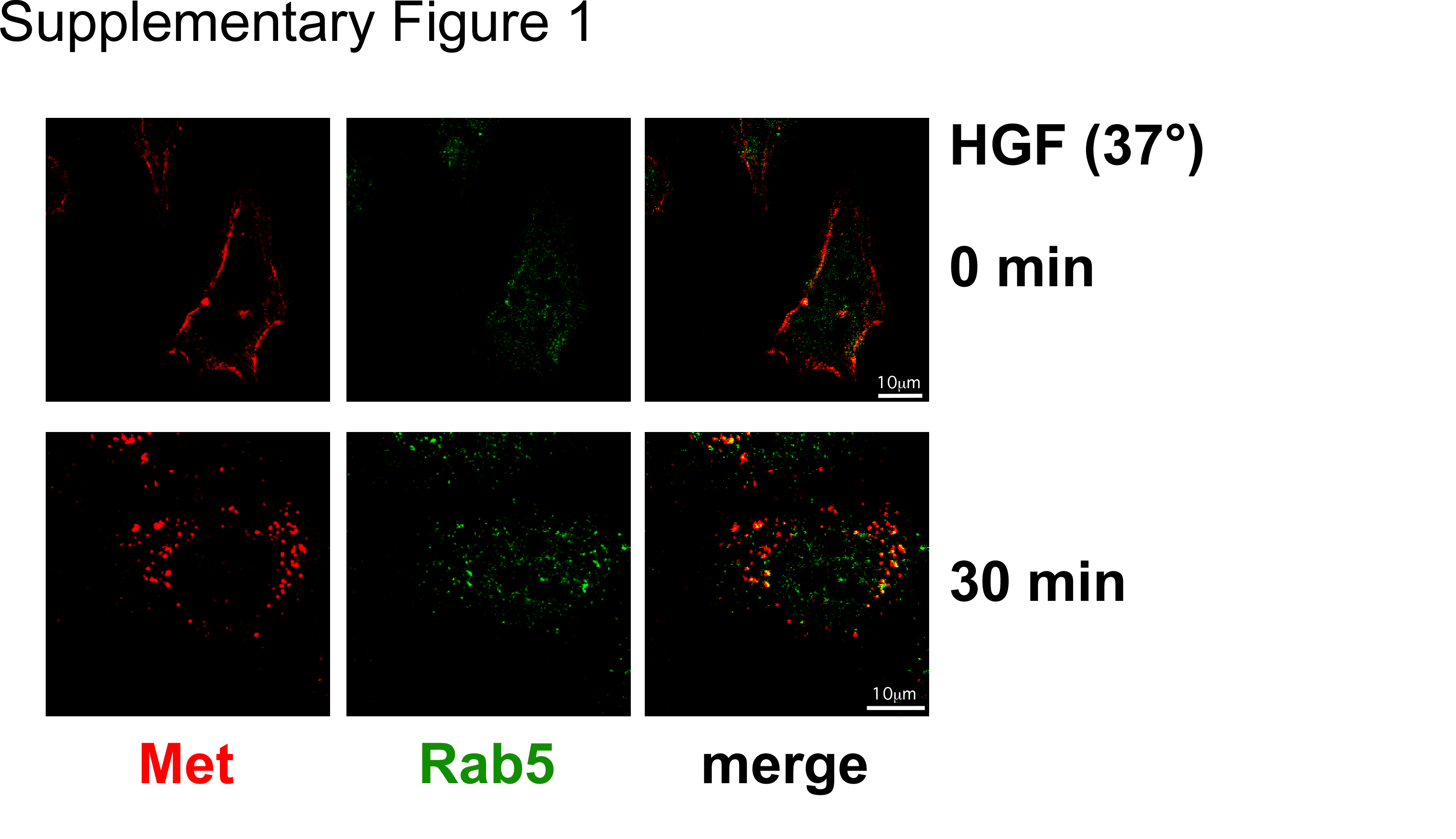

Supplement: Figure S1 — Colocalization of Met (Red) with endogenous Rab5 (green). (TIF) [file pone.0062357.s001.tif]

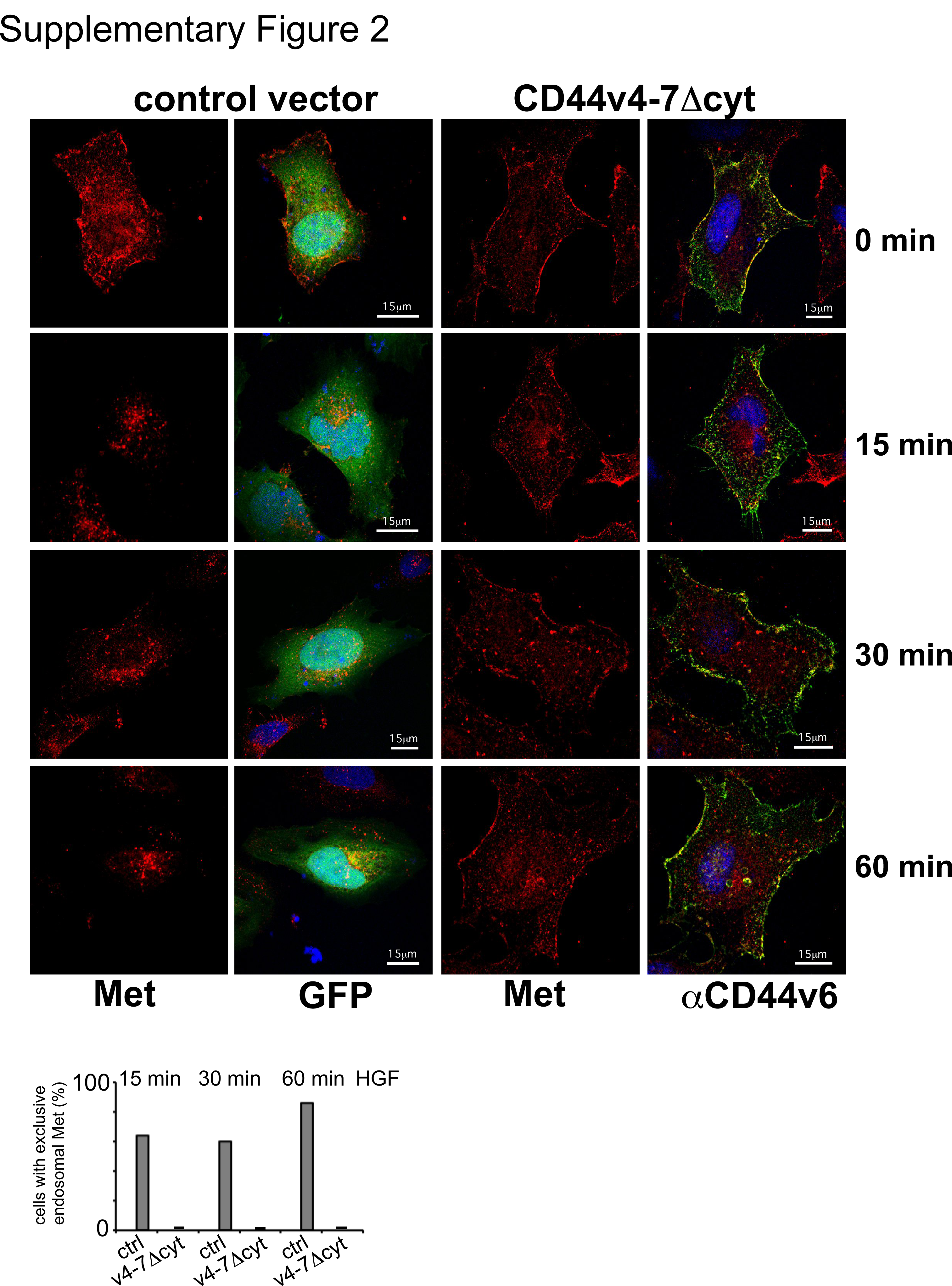

Supplement: Figure S2 — The experiment was performed as described in Figure 5B . In this case, the cells were transfected with a rat CD44v4-7Δcyt instead of human CD44v6Δcyt. (TIF) [file pone.0062357.s002.tif]
